# Region-Specific Expression Patterns of lncRNAs in the Central Nervous System: Cross-Species Comparison and Functional Insights

**DOI:** 10.3390/ijms262412069

**Published:** 2025-12-15

**Authors:** Tresa López-Royo, Elisa Gascón, Laura Moreno-Martínez, Sofía Macías-Redondo, Pilar Zaragoza, Raquel Manzano, Rosario Osta

**Affiliations:** 1 LAGENBIO, Faculty of Veterinary, University of Zaragoza, Miguel Servet 177, 50013 Zaragoza, Spain; tlopez@unizar.es (T.L.-R.);; 2 Centre for Biomedical Research in Neurodegenerative Diseases (CIBERNED), Instituto de Salud Carlos III, 28029 Madrid, Spain; 3 AgroFood Institute of Aragon (IA2), University of Zaragoza, 50013 Zaragoza, Spain; 4 Institute of Health Research of Aragon (IISA), 50009 Zaragoza, Spain; 5 Instituto Aragonés de Ciencias de la Salud (IACS), Centro de Investigación Biomédica de Aragón (CIBA), 50009 Zaragoza, Spain; 6 Recombinant Proteins Department, Certest Biotec S.L., Pol. Industrial Río Gallego II, Calle J, 1, San Mateo de Gállego, 50840 Zaragoza, Spain

**Keywords:** long noncoding RNAs (lncRNAs), CNS, spinal cord, brainstem, frontal cortex

## Abstract

Increasing evidence demonstrates that long noncoding RNAs (lncRNAs) are crucial for brain evolution and proper development and function of the central nervous system (CNS), exhibiting specific time-, spatial-, and sex-biassed expression patterns. This study investigated whether region-specific spatial expression patterns of brain-relevant lncRNAs are conserved between the mouse and human CNS. Demonstrating such cross-species conservation informs the translational value of mouse models for lncRNA biology. To test this, the expression of 14 lncRNAs was studied in the adult CNS of mice and humans across three different regions (spinal cord, brainstem, and frontal cortex), and age effects were assessed in mice. The results demonstrated conserved expression patterns between the two species, with region-specific changes. The frontal cortex exhibited high expression of *Meg3*, *Miat*, and *Pvt1* lncRNAs, while the spinal cord showed high levels of *Hotair* and *Gas5*. Additionally, *Malat1* displayed lower levels in females compared to males in the spinal cord compared to other regions. Finally, through GO functional enrichment analysis and literature review, this study emphasizes the role of lncRNAs in CNS physiology and disease, suggesting their involvement in neurological processes and conditions such as cortical development, neuronal synapsis, schizophrenia, Alzheimer’s, Parkinson’s, and amyotrophic lateral sclerosis. Overall, this research highlights the importance of further investigating the role of lncRNAs in brain function and their potential as key players in neurological disorders, opening the door to explaining the high region- and sex-specific effects of these disorders.

## 1. Introduction

The human genome produces thousands of long noncoding RNAs (lncRNAs), transcripts exceeding 200 nucleotides in length that lack evident protein-coding potential [1]. lncRNA transcripts share some similarities with messenger RNAs as they are typically transcribed by RNA polymerase II, exhibit classical splice sites, contain intron and exon structures, undergo alternative splicing, and share histone modifications with protein-coding genes [2]. However, lncRNAs have long been considered as “transcriptional noise” on the pretext that they do not code for proteins and their sequence is poorly conserved between species.

LncRNAs are found in a wide range of species, including bacteria, insects, worms, mammals, fish, birds, and plants. Interestingly, despite the traditional emphasis on the importance of coding RNAs in biology, the evolutionary complexity of species is correlated with its relative number of lncRNAs. For instance, in flies, humans, and mice, the number of protein-coding genes is relatively similar, with flies having 13,969 genes, mice 21,848, and humans 19,951. Conversely, the number of lncRNAs increases significantly in more evolved species: 2545 in flies, 13,186 in mice, and 17,948 in humans [3].

In evolutionary terms, it is now understood that the lack of conservation in the primary sequence of lncRNAs does not typically affect their function, which can be preserved across different species. In fact, lncRNA function mainly relies on specific secondary structures that allow interaction with proteins and facilitate binding between DNA and RNA based on base pair complementarity [4,5]. Moreover, each lncRNA is not restricted to a single function. Instead, lncRNAs usually exhibit multiple interactions with different molecules, thus exerting several functions, which can also be highly tissue-specific. LncRNAs expressed in the brain show the strongest evolutionary conservation as compared to those expressed in other tissues [6,7,8], which has also been associated with increasing levels of brain complexity [9].

At the molecular level, major functions of lncRNA comprise the regulation of protein-coding gene expression at epigenetic, transcriptional, post-transcriptional, and translational levels. They also participate in transcriptional activity modulation, X-chromosome silencing, genome imprinting, chromatin modification, structural cell compartment formation, and many other biological processes [10,11,12,13,14].

At the cellular level, they have been shown to play a role in development, cell cycle regulation, and differentiation. In particular, lncRNAs are they are particularly abundant in the central nervous system (CNS), where a remarkable diversity can be found. In fact, an increasing number of lncRNAs have been confirmed to play crucial roles in evolution, adaptability, maintenance, differentiation, and operation of various neuronal subtypes [11,15,16]. Furthermore, lncRNAs expression pattern changes during neural stem cell differentiation, with a significant portion being brain-specific. For example, studies show that lncRNAs like *Dlx1as* and *Six3os* are required for specifying the neuronal and oligodendrocyte lineages, respectively [17,18].

Overall, an accurate programme of time and location-specific lncRNA expression is essential for the morphogenesis and function of the distinct CNS regions and cell types. Indeed, research in primates has demonstrated spatial-, age-, and sex-biassed changes in brain lncRNA expression, suggesting that these molecules may constitute a regulatory system that potentially contributes to brain development, ageing, and evolution [19,20]. Despite their importance, and the RNA-seq studies and single-cell analyses performed over the last decade, the specific lncRNAs expressed in distinct brain regions.

To understand the expression and relevance of lncRNAs across anatomical and functional regions of the CNS, the conservation of region-specific spatial expression patterns between mouse and human was assessed, thereby informing the translational value of mouse models. For this purpose, lncRNA expression was evaluated in the spinal cord, brainstem, and frontal cortex of mice, observing specific regional enrichment of certain lncRNAs. In addition, lncRNAs from human brainstem and frontal cortex samples were analyzed, evidencing that lncRNA expression profiles persisted across species. Finally, a functional enrichment study was carried out to investigate the relevance of lncRNAs highly expressed in each area of the CNS and their putative implication in CNS main biological functions and neurological disorders.

## 2. Results

### 2.1. Constitutive lncRNA Expression Across the Murine Central Nervous System

To explore potential differences in lncRNA expression patterns, this study investigated the transcriptional levels of 14 lncRNAs in three areas of the CNS from adult mice. The selected transcripts (*Meg3*, *Hotair*, *Malat1*, *Gas5*, *Neat1*, *Myhas*, *Xist*, *CDR1os*, *Snhg1*, *Snhg16*, *Miat*, *Pvt1*, and *H19*) have previously been identified as brain-expressed in RNA-seq studies [21,22,23], show moderate-to-high conservation between mammals, and are known to be involved in key CNS-related functions and pathologies. Given its recent detection in the CNS and the lack of prior characterization, Myoparr was also included for exploratory analysis [24]. Expression profiles were assessed by real-time PCR in the spinal cord, brainstem, and frontal cortex at three postnatal ages (P60, P90, and P120).

**Figure 1:**
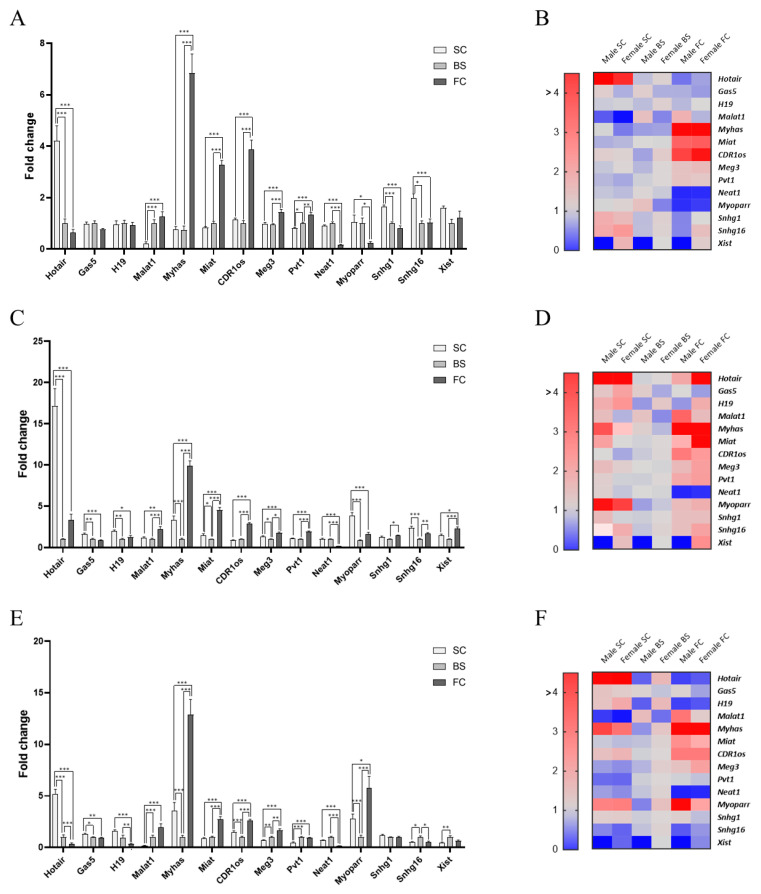
LncRNA constitutive expression in the spinal cord, brainstem, and frontal cortex from mice. (**A**,**C**,**E**) Transcript levels of the 14 lncRNAs studied in the spinal cord (SC, white bars), brainstem (BS, light grey), and frontal cortex (FC, dark grey) from mice at P60 (**A**), P90 (**C**), and P120 (**E**) are shown. Bar plots illustrate region-specific differences in relative expression levels across CNS regions, independent of sex. Fold change values show the expression relative to the brainstem (set to 1) at each age. Each data point represents the mean ± SEM of twelve (spinal cord) or ten (brainstem and frontal cortex) sex-balanced mice (Xist data correspond only to female mice). Asterisks denote a Student’s t-test *p*-value < 0.05 (*), <0.01 (**), <0.001 (***). (**B**,**D**,**F**) Heat map of lncRNA expression in the spinal cord, brainstem, and frontal cortex of male and female mice at P60 (**B**), P90 (**D**), and P120 (**F**). Heatmaps consider sex-specific variations while showing these regional expression patterns.

The results showed the differential expression of lncRNAs across the different regions of the CNS (Figure 1). Interestingly, the proportion of lncRNAs showing regional differences/disparities increased with age. Specifically, at P60, 79% of the lncRNAs exhibited significant variation among the distinct/studied CNS regions, as compared to 100% and 93% at P90 and P120, respectively. These differences were assessed independently of sex and are shown in the bar plots (Figure 1A,C,E).

Surprisingly, only four of the lncRNAs studied (*Xist*, *Myoparr*, *Snhg1*, and *Snhg16*) showed dynamic fluctuations across the different ages with no clear general trend, while the others exhibited consistent region-specific expression patterns over time.

For instance, in the spinal cord, the transcriptional levels of *Hotair* (all ages), *Gas5,* and *H19* (P90, P120) were higher than in the brainstem and frontal cortex (Figure 1C–F). Conversely, *Malat1* was significantly lower in this tissue (Figure 1A–F). Similarly, in the frontal cortex, *Meg3*, *CDR1os*, *Miat*, and *Pvt1* levels were particularly abundant, whereas *Neat1* levels were relatively low (Figure 1A–F). Moreover, *Myhas* expression was increased in the cortex as compared to the spinal cord and brainstem, and it was also significantly diminished in the brainstem versus the spinal cord (Figure 1A–F).

No sex-related differences were found in *Neat1*, *Myhas*, *Myoparr*, and *Pvt1* levels across the examined regions. On the other hand, the expression of *Meg3*, *Hotair*, *Malat1*, *Gas5*, *Xist*, *CDR1os*, *Snhg1*, *Snhg16*, *Miat*, and *H19* varied between male and female samples (Figure 1B,D,F and Table S3). Overall, differences varied depending on the tissue type and age. However, two lncRNAs exhibited consistent sex-biassed expression patterns: *Xist*, which is known to be exclusively expressed in females due to its role in X-chromosome inactivation, and *Malat1*, which showed higher expression levels in males in every tissue and at all examined ages. To our knowledge, this is the first report describing a sex-related expression bias for *Malat1* in the CNS.

Overall, when sex differences were present, higher lncRNA expression was detected in the spinal cord of males, whereas females showed higher levels in the frontal cortex and brainstem. Regarding tissue distribution, the frontal cortex displayed the largest number of lncRNAs differentially expressed between sexes (10), followed by the spinal cord (9) and brainstem (8). In terms of age, P90 showed the highest number of lncRNAs differentially expressed between the sexes (11 vs. 9 and 8).

### 2.2. Conservation of lncRNA Expression Patterns Between Mouse and Human

To verify the conservation of lncRNA expression patterns between species across CNS regions, post-mortem human tissue samples were also analyzed (Table 1). Of the 14 lncRNAs tested in mice, 9 were successfully detected in brainstem and frontal cortex human samples, including *MEG3*, *MALAT1*, *GAS5*, *NEAT1*, *SNHG1*, *SNHG16*, *MIAT*, *PVT1*, and *H19*.

**Figure 2:**
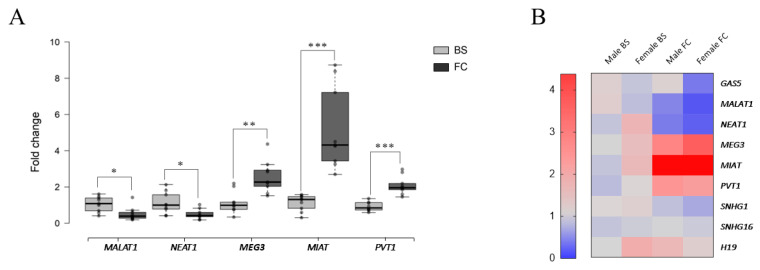
LncRNA differential expression in human brainstem and frontal cortex. (**A**) LncRNA constitutive transcript levels of *MALAT1*, *NEAT1*, *MEG3*, *MIAT*, and *PVT1* in brainstem (BS, light grey) and frontal cortex (FC, dark grey) from post-mortem samples of patients. Box plots depict significant differences in relative expression levels between both regions, disregarding sex. Relative expression refers to the brainstem. Results are shown as mean ± SEM. Asterisks denote student *t*-test *p*-value < 0.05 (*), <0.01 (**), <0.001 (***). (**B**) Heat map of lncRNA expression in human samples. Heat maps show separate data for males and females.

Among these, five lncRNAs showed significant differences between both regions (Figure 2 and Figure S1). In particular, *MALAT1* and *NEAT1* exhibited higher expression in the brainstem, while *MEG3*, *MIAT*, and *PVT1* showed increased expression in the frontal cortex. Altogether, these results were consistent with those obtained in mice.

These findings were further validated for *MALAT1*, *NEAT1*, *MEG3*, and *MIAT* using microarray data obtained from six additional brains documented in [25] (see Figure S2).

Gender-related differences were only statistically significant for *GAS5* and *MIAT* in the cortex and brainstem, respectively. As compared to mouse samples, this decrease could be due to the limited N (n = 6 males; n = 3 females) and the high variability intrinsic to human patient samples.

### 2.3. Functional and Enrichment Bioinformatic Analysis of lncRNAs Across CNS Regions

To better understand the tissue-specific enrichment and potential functions of lncRNAs within each CNS region, we initially performed a Gene Ontology (GO) functional and enrichment bioinformatic analysis on lncRNAs. Due to the limited availability of data, the analyses produced sparse results, with most lncRNAs lacking annotations. Even those that were annotated provided limited information, typically related to the broad aspects of RNA metabolism regulation. To overcome this limitation, we conducted a GO analysis of lncRNA interactomes to better understand their functional roles, using the EVLncRNAs V3.0 database to retrieve their reported molecular interactions with protein-coding genes (mRNAs), genomic DNA, and genes targeted by miRNAs.

**Figure 3:**
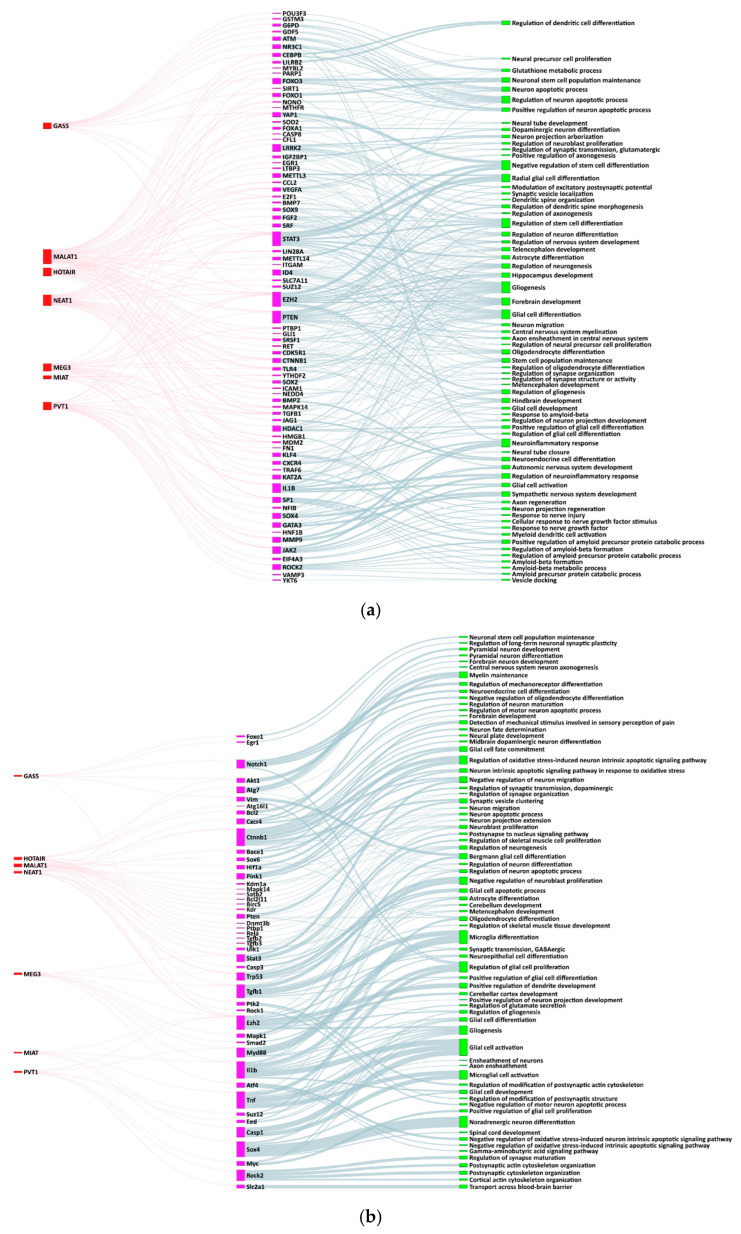
Sankey diagram of lncRNA interactomes. This diagram visualizes the relationships of specific lncRNAs (*Gas5*, *Hotair*, *Malat1*, *Meg3*, *Miat*, *Neat1*, and *Pvt1*) with genes and relevant biological processes in the CNS: (**a**) human and (**b**) mice.

Therefore, we analyzed the interactomes of seven lncRNAs that had exhibited a consistent pattern of differential region enrichment (*Gas5*, *Hotair*, *Malat1*, *Neat1*, *Meg3*, *Miat*, and *Pvt1*) using GO analysis. The results obtained from both human and mouse species are provided in the Supplementary Data files. Among all the results from this functional analysis, the biological processes most closely related to the central nervous system are highlighted in Figure 3 and detailed in Tables S4 and S5.

For all three lncRNAs up-regulated in the frontal cortex (*Miat*, *Meg3*, and *Pvt1*), GO analysis revealed associations with glia-related biological processes, such as gliogenesis, glial cell activation, development and differentiation, neuronal ensheathment (the process of glial cells wrapping around neuronal axons), and neuroinflammatory response. Additionally, individually, *Meg3* was related to forebrain and hippocampus development, neuron migration, and axon regeneration, whereas *Pvt1* was associated with regulation of synapse maturation, amyloid-beta metabolic processes, and postsynaptic and cortical actin cytoskeleton organization, among others. Interestingly, despite the higher expression of *Miat* in the frontal cortex, GO analysis revealed a stronger association of this lncRNA with biological processes pertinent to the brainstem and spinal cord mediated through the *Ezh2* and *Sox4* genes (noradrenergic neuron differentiation, cerebellar cortex, and spinal cord development).

In the case of NEAT1, which is decreased in the cortical region, the biological processes of interest found in humans were telencephalon and metencephalon development, glial and neuroendocrine cell differentiation, response to amyloid-beta, glutathione metabolic process, neuronal apoptosis, and regulation of synapse organization. In mice, the identified processes are primarily related to the regulation of neuronal and glial survival and apoptosis—particularly under oxidative stress—the development and differentiation of neurons (including dopaminergic neurons), and the modulation of synaptic plasticity and sensory pain perception.

*Gas5*, which is increased in the spinal cord, was associated with differentiation and proliferation of different cell types in the nervous system (Bergmann glia, astrocytes, oligodendrocytes, neuroendocrine cells, and mechanoreceptors), the maintenance and regulation of stem cells and neuronal precursors, the control of apoptosis and synaptic plasticity, as well as associated functions such as myelin maintenance, neuroinflammatory response, and glutathione metabolism. Hotair, also enriched in this region, was related to general development, maintenance, and function processes of the CNS, such as neuron arborization, axonogenesis, synaptic transmission, or proliferation and differentiation of neurons and glial cells. In addition, it was also associated with motor neuron apoptosis, which may partly account for its enrichment in the spinal cord. Nevertheless, it was likewise linked to pathways that are predominantly brain-specific and have minimal relevance to the spinal cord, including the development of the hippocampus, telencephalon, and forebrain.

Finally, according to our bioinformatic analysis, Malat1 -which is reduced in the spinal cord- was mainly involved in regulating the differentiation of glial, oligodendrocyte, and dendritic cells; the proliferation and migration of neuroblasts; and the extension of neuronal projections with dendritic spine formation, as well as axonal myelination and ensheathment. In addition, it was also related to the development of the forebrain, hindbrain, cerebellum, and metencephalon; neuronal apoptosis and the response to oxidative stress; the regulation of synaptic organization, and GABAergic transmission.

## 3. Discussion

The CNS exhibits large biological complexity, being responsible for the regulation of cognitive, emotional, and physiological functions. Understanding its regulation and balance thus remains intricate, yet extremely important.

In this context, lncRNAs regulate essential biological processes in the brain, such as neural cell differentiation, neurite outgrowth, or synapse regulation and function [11,15,16,26]. Indeed, abnormal lncRNA expression has been associated with devastating neurological diseases, including glioma [27,28], schizophrenia [29], Alzheimer’s (AD) and Parkinson’s (PD) diseases [30,31,32,33,34], developmental delay [35], and autism [36].

Many of these disorders involve specific processes, brain areas, and cell types. Likewise, gender influences disease incidence and development. Understanding the causes underlying this specificity will shed light on the pathogenesis of these disorders and unravel potential therapeutic targets. In this sense, lncRNAs, which are highly specific molecules that regulate gene expression and show distinct abundance and functions according to factors such as sex, age, and cell type, emerge as key candidates for understanding the selective processes triggered in these pathological conditions.

To this end, this work evaluates the expression of 14 lncRNAs, selected based on a literature review for their established roles in CNS development and function, in three different regions of the mouse CNS, namely the spinal cord, brainstem, and frontal cortex. Results showed differential lncRNA expression among regions, particularly between the anatomically distant spinal cord and frontal cortex. Specifically, *Meg3*, *CDR1os*, *Miat*, *Pvt1*, and *H19* were up-regulated in the frontal cortex compared to the spinal cord and brainstem, while *Neat1* was down-regulated. On the other hand, in the spinal cord, *Hotair* and *Gas5* levels were augmented, and *Malat1* expression diminished. Finally, only *Myhas* was significantly reduced in the brainstem. LncRNA expression was also assessed in post-mortem brainstem and frontal cortex samples from human patients, proving similar expression patterns, which highlights the use of mice as a model for neurological disorders in translational research and evidences the biological relevance of these lncRNAs.

The regional patterns observed could be explained by intrinsic differences in the cellular composition and functional and molecular requirements of each region of the CNS. To further investigate the possible causes of local lncRNA enrichment, functions and biological relevance of these lncRNAs were investigated through GO functional analysis of their interactomes and literature review.

Notably, lncRNAs enriched in the frontal cortex were linked to processes such as forebrain development, glial regulation and differentiation, neurogenesis, axonal regeneration, and both synaptic and post-synaptic signalling. Their evolutionarily conserved enrichment in this region may reflect the critical role these processes play in supporting the frontal cortex’s complex cognitive functions—including planning, decision-making, organization, working memory, cognitive flexibility, emotional regulation, social behaviour, and voluntary movement control—which demand high levels of neuroplasticity, structural reorganization, synaptic complexity, and robust glia–neuron interactions.

Additionally, several of these lncRNAs—*Meg3*, *Pvt1*, and *Malat1* (with MALAT1 showing reduced expression in the spinal cord)—are involved in GABAergic signalling, which is essential for proper cortical development and implicated in disorders with cortical affection such as age-related cognitive decline, schizophrenia, and autism [37,38,39]. Similarly, *Pvt1* plays a role in β-amyloid metabolism; β-amyloid aggregates in the cortex are a characteristic hallmark of Alzheimer’s disease-related dementia [40]. In contrast, lncRNAs with lower expression in the cortex but enriched in the spinal cord—*Neat1*, *Gas5*, and *Hotair*—were implicated in processes such as myelination, motor neuron apoptosis, and glutathione metabolism. While these processes are relevant throughout the nervous system, they hold particular significance in the spinal cord due to its unique composition and functions. Notably, they represent key canonical events in ALS, a disease characterized by predominant spinal cord involvement [41,42].

Furthermore, certain lncRNAs showed significantly different expression profiles between males and females within the same CNS area. In some cases, these differences were age- and tissue-dependent (*Meg3*, *Hotair*, *Gas5*, *CDR1os*, *Snhg1*, *Snhg16*, *Miat*, and *H19*), whereas in the case of *Xist* and *Malat1*, these changes were consistent across ages and regions. *Xist* has been widely reported to be expressed only in individuals with two X chromosomes. However, to the best of our knowledge, this is the first time that a sexual dimorphism is reported for *Malat1* in the CNS, which could be relevant in neurological disorders with a marked gender influence, such as schizophrenia, depression, AD disease, or ALS.

Nevertheless, and despite the discussion above, some lncRNAs are functionally linked to broad processes affecting the entire nervous system, or even to processes that do not precisely align with the regions where they are enriched. This may reflect limitations in current annotations or indicate indirect effects and systemic regulatory roles that extend beyond local functions. Furthermore, we acknowledge that this study relies on bulk tissue analysis, which provides an averaged expression level across a heterogeneous cell population. This approach, while robust for identifying regional patterns, inherently limits our ability to resolve cell-type-specific expression changes or complex regulatory dynamics, such as those that might occur within neurons, astrocytes, or microglia independently. Future studies using single-cell resolution (scRNA-seq) [43] will be essential to dissect the specific cellular contributions to the lncRNA signatures we have identified.

In brief, these findings highlight the complex role of lncRNAs in regulating crucial biological processes in the brain and spinal cord. A better understanding of the distribution and functions of lncRNAs in the central nervous system may offer valuable insights into CNS functioning and the pathogenic mechanisms underlying neurological and neurodegenerative diseases. Moreover, the conserved, region-specific lncRNA programmes observed across mouse and human indicate preserved regulatory logic and support the translational relevance of murine CNS models, providing a functional anchor to interrogate lncRNA mechanisms, biomarkers, and therapeutic candidates in neurological disease

## 4. Materials and Methods

### 4.1. Human Sample Collection

Brain tissue samples and data from patients included in this study were collected, processed, and provided by the CIEN Tissue Bank (CIEN Foundation, Instituto de Salud Carlos III) and Biobanco en Red de la Región de Murcia (BIOBANC-MUR, registration number from the Registro Nacional de Biobancos B.0000859), following standard operating procedures with appropriate approval of the Ethical and Scientific Committees. All subjects provided written informed consent, and El Comité de Ética de la Investigación de la Comunidad de Aragón (CEICA) (Ref. PI17/0025, updated on June 2023) and El Comité Científico del banco de tejidos de la Fundación CIEN (Ref. CCS17003, updated CEI PI 79_2023) approved this research. All material was released to the investigators fully anonymized; therefore, only the brain region, sex, and age were available for each donor. Medication use, lifestyle factors, and detailed clinical histories were not provided by the repositories. For detailed clinical characteristics, please refer to Supplementary Table S1.

### 4.2. Animals

Wild-type B6SJL mice were purchased from Janvier Labs and housed at the animal facilities of the Centro de Investigación Biomédica de Aragón in a pathogen-free environment and under a standard light/dark (12:12) cycle. Food and water were provided ad libitum.

The care and use of animals adhered strictly to the Spanish Policy for Animal Protection RD53/2013, in compliance with the European Union Directive 2010/63 regarding the safeguarding of animals used for experimental and scientific purposes. All experimental protocols received approval from the Ethics Committee for Animal Experiments at the University of Zaragoza and were registered with code numbers PI29/13 and PI08/19.

### 4.3. Mice Sample Collection

Spinal cord, brainstem, and frontal cortex samples were collected from ten to twelve sex-matched mice at different stages: 60, 90, and 120 days of postnatal life (P60, P90, and P120). Samples were harvested after CO_2_ euthanasia, frozen in dry ice, and stored at −80 °C until processed.

### 4.4. RNA Extraction

For mouse tissue, the samples were homogenized in Trizol Reagent using Tissue Lyser LT (Qiagen; Hilden, Germany). Total RNA was isolated using Direct-zol^TM^ RNA MiniPrep Kit (Zymo Research; Irvine, CA, USA), according to the manufacturer’s instructions.

For human brain samples, RNA purification was performed as previously described in Oros et al., 2017 [44]. The quality and concentration of each extraction were measured with a Nanodrop ND-1000 spectrophotometer (Thermo Fisher Scientific; Waltham, MA, USA), and integrity was reported by the biobanks as RQI. As expected for post-mortem CNS tissue, some variability in RQI was observed and was managed through assay design and housekeeping-normalized ΔΔCt analyses.

### 4.5. Real-Time PCR

For lncRNA quantification, cDNA was synthesized using the High-Capacity cDNA Reverse Transcription Kit from Applied Biosystems (Thermo Fisher Scientific; Waltham, MA, USA). Reverse transcription quantitative PCR (RT-qPCR) was conducted from diluted cDNA in triplicate using the Quant Studio^TM^ 3 Real Time PCR Instrument from Applied Biosystems (Thermo Fisher Scientific). Custom self-designed Syber Green Primers (Thermo Fisher Scientific) employed in this work are detailed in Table S2.

The relative gene expression was calculated by the 2^−∆∆CT^ method as described by Livak & Schmittgen [45]. In mouse samples, *Gapdh* and *Actb* served as housekeeping genes, while GAPDH was used to normalize human samples.

### 4.6. Functional Enrichment Study

To perform the functional enrichment study, a list of all genes and proteins related to each lncRNA was first made using the information present in the EVLncRNAs V3.0 database (https://www.sdklab-biophysics-dzu.net/EVLncRNAs2/ (acessed on 9 April 2025)) [46]. Once this information was obtained, the functional enrichment analysis of genes was carried out with the RStudio (v4.4.1) environment [47]. The Bioconductor R package, clusterProfiler (v4.8.2) [48] was used with default statistical thresholds and the organism (OrgDb) set to “org.Hs.eg.db” and “org.Mm.eg.db”. clusterProfiler is a popular package renowned for its ability to perform comprehensive functional and pathway enrichment analyses, allowing for the analysis and visualization of enrichment across numerous organisms. This analysis specifically focused on Gene Ontology (GO) terms, categorizing them into (1) biological processes, (2) molecular functions, and (3) cellular components. GO scores with a *p*-value < 0.05 were considered statistically significant. The networkD3 package (v0.4.1) was utilized to make the Sankey diagram [49].

### 4.7. Statistical Analysis

The results are shown as the mean value ± the standard error of the mean (SEM) or the standard deviation (SD), as indicated. To establish significant differences between CNS areas, one-way ANOVA and Student’s t-tests were performed when comparing three (in mice) and two (in humans) different tissues, respectively. Outliers were detected by the iterative Grubb’s test and excluded from the analysis. GraphPad Prism software (version 8.0.1) was used for the statistical analysis. Differences were considered statistically significant if *p* < 0.05 (*) and highly significant if *p* < 0.01 (**), *p* < 0.001 (***).

## 5. Conclusions

This work contributes to the understanding of the differential expression and function of lncRNA across distinct areas of the central nervous system, demonstrating similar patterns between mouse and human and evidencing gender and age influence on lncRNA levels.

The evolutionary conservation in lncRNA expression, along with the association of lncRNAs with crucial roles in CNS development and function, supports the hypothesis that lncRNA function is conserved across species despite the lack of primary structure conservation. However, further studies are needed to investigate the reasons for specific lncRNA enrichment in determined brain regions and to clarify the roles of lncRNAs in the central nervous system function, development, and disease, a field that remains poorly understood.

## Figures and Tables

**Table 1:** General characteristics of the patient cohort.

		Tissue	
Parameter		Brainstem (N = 9)	Frontal Cortex (N = 9)
Gender (n)	Male	6 (66.67%)	6 (66.67%)
	Female	3 (33.33%)	3 (33.33%)
Age		58.78 ± 10.56	59.11 ± 10.55

## Data Availability

The datasets produced and analyzed during the present study can be obtained from the corresponding author upon reasonable request.

## Supplementary Materials

The supporting information can be downloaded at: https://www.mdpi.com/article/10.3390/ijms262412069/s1.
