# Supplementary material for: Region-Specific Expression Patterns of lncRNAs in the Central Nervous System: Cross-Species Comparison and Functional Insights
Source: Int J Mol Sci. 2025 Dec 15;26(24):12069. doi: 10.3390/ijms262412069 (PMC12733350; doi:10.3390/ijms262412069)
Supplement: Supplementary file 1 [file ijms-26-12069-s001.zip › ijms-3960821-supplementary.pdf]

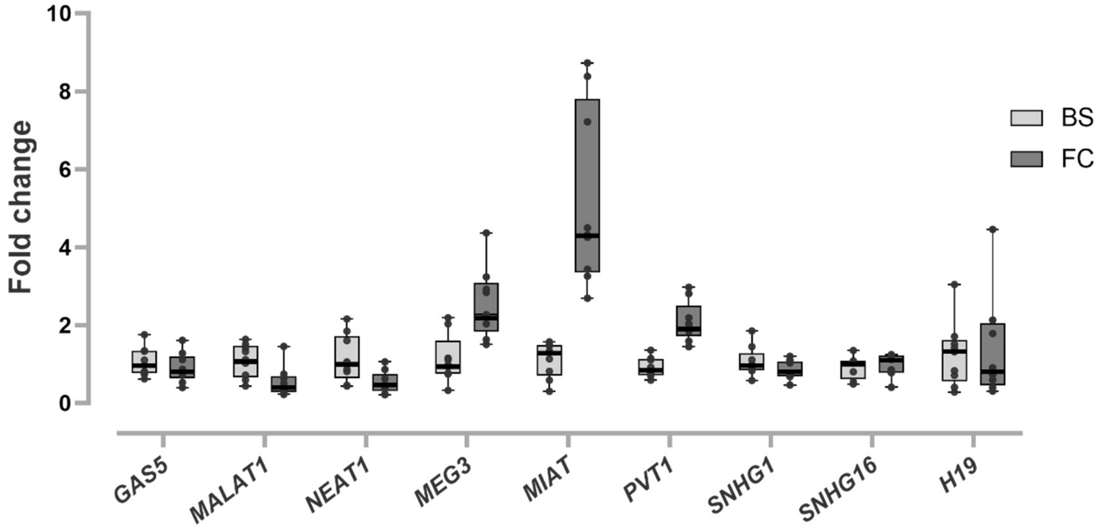

Figure S1. LncRNA constitutive expression in human brainstem and frontal cortex. Transcript levels of GAS5, *MALAT1*, *NEAT1*, *MEG3*, *MIAT*, *PVT1, SNHG1, SNHG16 and H19* in brainstem (BS, light grey) and frontal cortex (FC, dark grey) from *post mortem* samples of patients. Relative expression is referred to brainstem (set as 1). Results are shown as mean ± SEM. p value <0.05 (*), <0.01 (**), <0.001 (***).

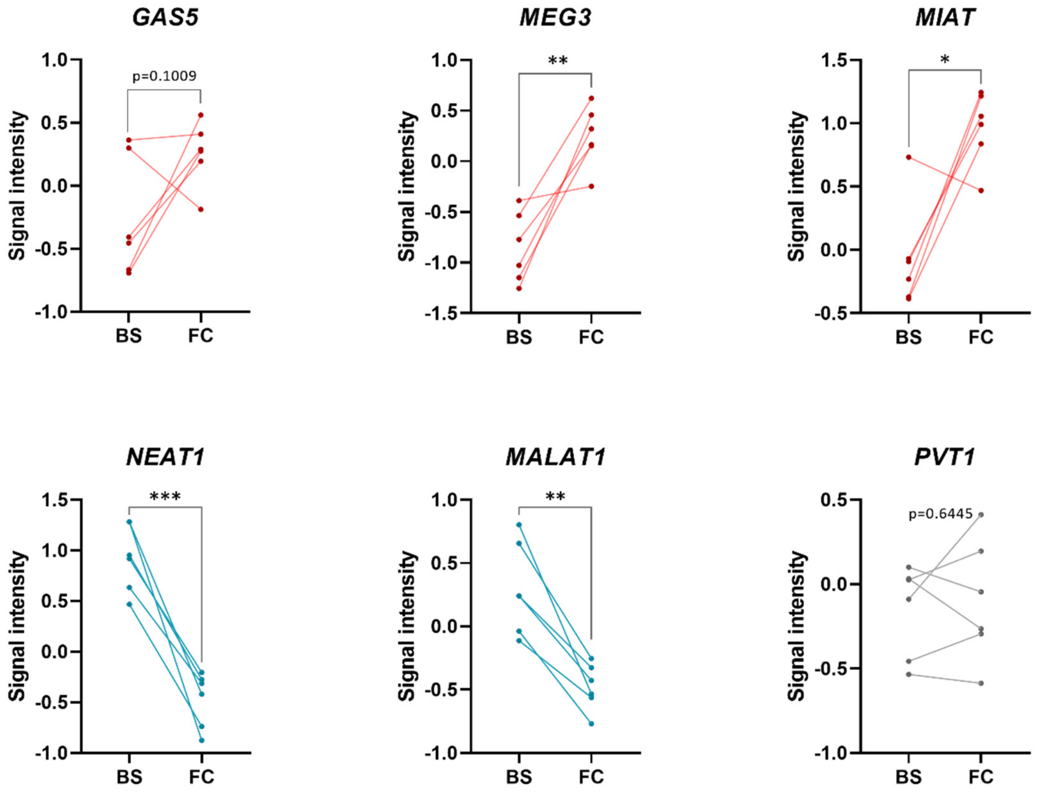

Figure S2. Microarray-based analysis of gene expression for *GAS5*, *MEG3*, *MIAT*, *NEAT1*, *MALAT1*, and *PVT1* in the brainstem and frontal cortex. Expression levels of these lncRNAs were derived from normalized data from the Human Brain Microarray Study in the Allen Brain Atlas (N=6, male: 5, female: 1, white or Caucasian: 3, Hispanic:1, black or African American: 2). For the frontal cortex, data corresponding to top level structure name “frontal lobe” (4009) were used; for the medulla oblongata, data for “basal part of pons” (9135) and “pontine tegmentum” (9135) were used. When multiple probes were available for a given gene, the probe with the highest intensity value was selected (selected probes: *GAS5* 1022367, *MEG3* 1010763, *MIAT* 1015344, *NEAT1* 1022171, *MALAT1* 1022437, *PVT1* 1019779). Statistical analyses were performed in GraphPad Prism using t-tests for paired data to compare tissues. Significance thresholds: p value <0.05 (*), <0.01 (**), <0.001 (***).

Table S1. Detailed data from study patients's cohort. RQI: RNA Quality Indicator (range 0 to 10); NI: no information available.

Patient	Gender	Age	Brain Areas	RQI value		Death cause
				BS	FC	
1	Male	43	Brainstem, Frontal Cortex	3.7	NI	NI
2	Male	53	Brainstem, Frontal Cortex	NI	NI	Rectal adenocarcinoma
3	Male	57	Brainstem, Frontal Cortex	NI	NI	Lung carcinoma
4	Male	58	Brainstem, Frontal Cortex	NI	NI	Brain hemorrhage
5	Male	58	Brainstem, Frontal Cortex	NI	NI	Ischemic cardiopathy
6	Male	56	Brainstem	4.3	NI	NI
7	Male	63	Frontal Cortex	NI	5.6	NI
8	Female	83	Brainstem, Frontal Cortex	5.6	5.7	NI
9	Female	59	Brainstem, Frontal Cortex	3.0	3.4	NI
10	Female	62	Brainstem	6.7	NI	NI
11	Female	58	Frontal Cortex	NI	NI	Acute subdural hematoma

Table S2. Primers used for qRT-PCR. This table includes the following information for each primer pair: primer sequences, ID target lncRNA sequence and organism, amplicon size and melting temperature (Tm).

LncRNA or Gene	Sequence	Organism	NCBI ID target gene sequence	Amplicon Size	Tm (°C)
**ACTB**	F: 5'-AGAGGGAAATCGTGCGTGAC-3' R: 5'-CAATAGTGATGACCTGGCCGT-3'	*Mus musculus*	NM_007393.5	138 bp	60.00
**CDR1os**	F: 5'-CACACTGAAGGTCAGCACGA-3' R: 5'-TTCACAAGTCCTCGATGCCAA-3'	*Mus musculus*	NR_030706.1	101 bp	60.00
**GAPDH**	F: 5'-AGGTCGGTGTGAACGGATTTG-3' R: 5'-GGGGTCGTTGATGGCAACA-3'	*Mus musculus*	NM_001289726.2, NM_008084.4, NM_001411840.1	95 bp	60.00
	F: 5'-AGGTCGGAGTCAACGGATTTG-3' R: 5'-AGGGGTCATTGATGGCAACA-3'	*Homo Sapiens*	NM_001357943.2, NM_001289745.3, NM_001289746.2, NM_002046.7	96 bp	60.00
**GAS5**	F: 5'-AAAGGAGGATGAAGGCTTACG-3' R: 5'-TCACTGCATGTCCACTTGTC-3'	*Mus musculus*	NR_166055.1, NR_153814.2, NR_166052.1, NR_166051.1, NR_166050.1, NR_153815.2, NR_153813.2, NR_153812.2	89-308 bp	57.74
	F: 5'-TGCAGTGTGGCTCTGGATAG-3' R: 5'-ATGGCTTGAGTTAGGCTTGCT-3'	*Homo Sapiens*	NR_186289.1, NR_186257.1, NR_186272.1, NR_186275.1, NR_186273.1, NR_186282.1, NR_186268.1, NR_186270.1, NR_186285.1, NR_186281.1, NR_152533.2, NR_152526.2, NR_186263.1, NR_186283.1, NR_152524.2, NR_152530.2, NR_152521.2, NR_186264.1, NR_186262.1, NR_186278.1, NR_002578.4, NR_152534.2, NR_186279.1, NR_152525.2, NR_186287.1, NR_186286.1, NR_186260.1, NR_152528.2, NR_152527.2, NR_186274.1 , NR_152532.2, NR_186266.1, NR_186290.1, NR_186288.1, NR_186271.1, NR_186284.1, NR_186277.1, NR_152531.2, NR_186261.1, NR_186259.1, NR_186269.1, NR_186267.1, NR_186255.1, NR_186280.1, NR_186258.1, NR_152529.2, NR_152522.2, NR_186256.1, NR_186265.1, NR_152523.2	100 bp	60.00
**H19**	F: 5'-CAGAGCAAAGGCATCGCAAA-3' R: 5'-GCTCCCCTTTATCCGACCAG-3'	*Mus musculus*	NR_130974.1, NR_130973.1	144 bp	60.50
	F: 5'-TCAAGCCTGGGCCTTTGAAT-3' R: 5'-CCTTCCAGAGCCGATTCCTG-3'	*Homo Sapiens*	NR_002196.3, NR_185829.1, NR_131224.1, NR_131223.2	107 bp	60.00
**HOTAIR**	F: 5'-GGCTGCCTGAGTTCTTTTGC-3' R: 5'-TGCGGTGGAGATAGATGTGC-3'	*Mus musculus*	NR_047528.1	100 bp	60.00

	F: 5’-AAGCGAAGGGGTTGTGTAGG-3’ R: 5’-AAGGCTAGGGCTGGTTTCAC-3’	*Homo Sapiens*	NR_186241.1, NR_186242.1, NR_186243.1 , NR_186245.1, NR_186244.1, NR_186247.1, NR_047518.2, NR_186239.1, NR_186246.1, NR_003716.4, NR_047517.2, NR_186240.1	175 bp	60.00
**MALAT1**	F: 5’-CCCGAGGAAATCGCAGATAA-3’ R: 5’-CTCTGGTCAGCCTCCATTATAC-3’	*Mus musculus*	NR_002847.3	216 bp	57.74
	F: 5’-GCTCTGTGGTGTGGGATTGA-3’ R: 5’-GTGGCAAAATGGCGGACTTT-3’	*Homo Sapiens*	NR_144568.1, NR_144567.1, NR_002819.4	179 bp	60.00
**MEG3**	F: 5’-ATAGCGCCCCCTATTCATGC-3’ R: 5’-GGGAGCAGCTATGGATCACC-3’	*Mus musculus*	NR_003633.3, NR_027651.2	176 bp	60.00
	F: 5’-ATCCCGGACCCAAGTCTTCT-3’ R: 5’-CCCACATTCGAGGTCCCTTC-3’	*Homo Sapiens*	NR_046470.2, NR_046465.2, NR_046473.1, NR_003531.3, NR_046472.1, NR_046471.1, NR_046469.1, NR_046467.1, NR_046466.1, NR_046464.1, NR_033360.1, NR_033359.1, NR_033358.1, NR_003530.2, NR_002766.2	104 bp	60.00
**MIAT**	F: 5’-ACACCAACCCACAAGACCTG-3’ R: 5’-CGTGTGATGAGAACAGACGGA-3’	*Mus musculus*	NR_003718.2	167 pb	60.00
	F: 5’-CCACCGTAAGTAGACGTGGG-3’ R: 5’-TCCCCACACCCCTTTTCAAC-3’	*Homo Sapiens*	NR_185991.1, NR_033320.3, NR_185985.1, NR_185997.1, NR_033321.3, NR_185990.1, NR_185995.1, NR_185996.1, NR_185986.1, NR_033319.3, NR_185989.1, NR_185987.1, NR_185994.1, NR_185988.1, NR_185984.1, NR_185998.1, NR_003491.4, NR_185983.1, NR_185982.1, NR_185993.1	107 pb	60.00
**MYHAS**	F: 5’-CGTGCTGGATCTTGCGGAAT-3’ R: 5’-AGACAGCCAAGATGCACTGAC-3’	*Mus musculus*	NR_146138.1	125 bp	61.00
	F: 5’-TGTCCTTCCATCGTCAGCAC-3’ R: 5’-CTCTGGACTGGTGAGGGGTA-3’	*Homo Sapiens*	NR_125367.1	190 bp	60.00
**MYOPARR**	F: 5’-AAAGGGGTTGTCTTGGACGG-3’ R: 5’-TGGAGTGGTCCTGATGTGGT-3’	*Mus musculus*	NR_160520.1	143 bp	60.00
	F: 5’-AGGTCAACCACCAGGCATC-3’ R: 5’-TCTCTCTAGGGACCTGCTGAC-3’	*Homo Sapiens*	NR_160550.1	132 bp	60.00
**NEAT1**	F: 5’-TGGCCCCTTTTGTTCATTAGC-3’ R: 5’-TGGAAGGCCATTGTTTCAGG-3’	*Mus musculus*	NR_131212.1, NR_003513.3	112 bp	58.74

	F: 5'-GTGTTGTGCTAAACGCTGGG-3' R: 5'-GCCCAAACTAGACCTGCCAT-3'	*Homo Sapiens*	NR_131012.1, NR_028272.1	79 bp	60.00
**PVT1**	F: 5'-AGACAGACTTGCTCAGGTGAT-3' R: 5'-TCTAGGCACCTTTCCCAGTTT-3'	*Mus musculus*	NR_132747.1, NR_132746.1 NR_003368.2	105 bp	58.84
	F: 5'-GGGGAATAACGCTGGTGGAA-3' R: 5'-CAGCCACAGCCTCCCTTAAA-3'	*Homo Sapiens*		92 bp	60.00
**SNHG1**	F: 5'-TCCTGGTGACAAATCTCAGGC-3' R: 5'-TGGTACGGCTCCTTTGTTCTC-3'	*Mus musculus*	NR_002896.3	102 bp	60.00
	F: 5'-ACCTGCTTGGCTCAAAGGG-3' R: 5'-TTCAGCCTGGAGTGAACACAG-3'	*Homo Sapiens*	NR_003098.2, NR_152582.1, NR_152583.1, NR_152577.1, NR_152585.1, NR_152575.1, NR_152580.1, NR_152578.1, NR_152576.1, NR_152581.1, NR_152579.1, NR_152584.1	90 bp	60.00
**SNHG16**	F: 5'-GGCATAGGTAGATGGTGGGC-3' R: 5'-CCACTTAGCACACCCCTCAG-3'	*Mus musculus*	NR_027821.1, NR_027819.1	295 bp	60.00
	F: 5'-AGAGACCAAGGAGGGACTGT-3' R: 5'-TACTGGCACGAGGACAAAGC-3'	*Homo Sapiens*	NR_038110.1, NR_038108.1, NR_038111.1, NR_038109.1	71 bp	60.00
**XIST**	F: 5'-TCCAAGAGCGGGGAACTACT-3' R: 5'-TTCACAGTTGGTGGGAAGATGA-3'	*Mus musculus*	NR_001570.2	141 bp	60.00

Table S3. Sex-specific stadistically significant differences on lncRNA expression in mouse CNS.

LncRNA name	Sample	Average RNA fold change in Males	Average RNA fold change in Females	Difference between means	t-test p value	
**MEG3**	Mice P60 Brainstem	0,8545	1,022	0,1675 ± 0,06609	0,039	*
	Mice P90 Frontal cortex	1,541	1,992	0,4513 ± 0,1751	0,0328	*
	Mice P120 Brainstem	0,8366	1,163	0,3267 ± 0,1210	0,0271	*
	Mice P120 Frontal cortex	1,366	1,967	0,6014 ± 0,2352	0,0338	*
**HOTAIR**	Mice P90 Spinal cord	12,63	21,71	9,085 ± 3,338	0,0215	*
	Mice P90 Frontal cortex	1,85	4,769	2,919 ± 1,192	0,0401	*
	Mice P120 Brainstem	0,4508	1,549	1,098 ± 0,3058	0,0071	**
**MALAT1**	Mice P60 Spinal cord	0,4143	0,0256	-0,3887 ± 0,09184	0,0017	**
	Mice P60 Brainstem	1,384	0,6156	-0,7688 ± 0,1575	0,0012	**
	Mice P60 Frontal cortex	1,668	0,8488	-0,8189 ± 0,3139	0,0312	*
	Mice P90 Spinal cord	1,472	0,8316	-0,6404 ± 0,2229	0,0166	*
	Mice P90 Brainstem	1,355	0,6451	-0,7097 ± 0,1016	0,0001	***
	Mice P90 Frontal cortex	2,971	1,486	-1,485 ± 0,4364	0,0093	**
	Mice P120 Spinal cord	0,2593	0,06995	-0,1893 ± 0,04686	0,0029	**
	Mice P120 Brainstem	1,482	0,5179	-0,9643 ± 0,2011	0,0014	**
	Mice P120 Frontal cortex	2,708	1,227	-1,480 ± 0,4715	0,0138	*
**GAS5**	Mice P60 Spinal cord	1,17	0,8111	-0,3588 ± 0,06979	0,0004	***
	Mice P90 Spinal cord	1,315	1,949	0,6346 ± 0,2009	0,0102	*
	Mice P90 Brainstem	1,21	0,7897	-0,4205 ± 0,1442	0,0194	*
	Mice P120 Frontal cortex	1,093	0,7637	-0,3291 ± 0,04756	0,0001	***
	Human Frontal Cortex	1,081	0,556	-0,5250 ± 0,2023	0,0357	*
**NEAT1**	Mice P90 Frontal cortex	0,149	0,2067	0,05773 ± 0,02046	0,0224	*
**MYHAS**	Mice P60 Spinal cord	0,9917	0,5582	-0,4335 ± 0,1664	0,0314	*
**XIST**	Mice P60 Spinal cord	0	1,611	1,611 ± 0,07090	<0,0001	****
	Mice P60 Brainstem	0	1	1,000 ± 0,1668	0,0003	***
	Mice P60 Frontal cortex	0	1,212	1,212 ± 0,2700	0,002	**
	Mice P90 Spinal cord	0	1,445	1,445 ± 0,1871	<0,0001	****
	Mice P90 Brainstem	0	1	1,000 ± 0,05087	<0,0001	****
	Mice P90 Frontal cortex	0	2,288	2,288 ± 0,2208	<0,0001	****
	Mice P120 Spinal cord	0	0,4687	0,4687 ± 0,05993	<0,0001	****
	Mice P120 Brainstem	0	1	1,000 ± 0,1528	0,0002	***
	Mice P120 Frontal cortex	0	0,6316	0,6316 ± 0,09963	0,0002	***
**MYOPARR**	Mice P120 Brainstem	0,5989	1,401	0,8022 ± 0,2920	0,0252	*
**CDR1os**	Mice P60 Brainstem	0,7571	1,243	0,4858 ± 0,1544	0,0137	*
	Mice P90 Spinal cord	1	0,7786	-0,2216 ± 0,06511	0,0067	**
**SNHG1**	Mice P60 Brainstem	0,8836	1,116	0,2329 ± 0,09617	0,0418	*
	Mice P60 Frontal cortex	0,6168	1,012	0,3949 ± 0,1467	0,0274	*
	Mice P90 Spinal cord	1,534	0,9749	-0,5587 ± 0,2265	0,0333	*
**SNHG16**	Mice P60 Frontal cortex	0,6246	1,424	0,7992 ± 0,1640	0,0012	**
	Mice P90 Spinal cord	2,956	1,744	-1,213 ± 0,3312	0,0044	**

	Mice P90 Frontal cortex	1,419	1,94	0,5202 ± 0,2035	0,0339	*
	Mice P120 Frontal cortex	0,4082	0,6934	0,2852 ± 0,09084	0,0138	*
**MIAT**	Mice P60 Frontal cortex	2,954	3,625	0,6714 ± 0,2508	0,0281	*
	Mice P90 Spinal cord	1,983	1,015	-0,9676 ± 0,2301	0,0018	**
	Mice P90 Frontal cortex	3,869	5,191	1,322 ± 0,5125	0,0327	*
	Human Brainstem	0,914	1,517	0,6030 ± 0,2505	0,047	*
**H19**	Mice P90 Brainstem	0,7081	1,292	0,5839 ± 0,1487	0,0044	**
	Mice P90 Frontal cortex	0,706	1,782	1,076 ± 0,2927	0,0063	**
	Mice P120 Brainstem	0,4246	1,541	1,116 ± 0,3967	0,026	*

Table S4. Selection of main LncRNA-mediated Gene-Process Interactions related to the CNS in human. The processes for each lncRNA are ranked in descending order by their Fold Enrichment value.

LncRNA name	Biological Process	Fold Enrichment	*p-value*	Gene
GAS5	Negative regulation of stem cell differentiation	72.64	9,170E-06	EZH2/STAT3/YAP1
	Neuronal stem cell population maintenance	52.46	6,586E-04	FOXO1/FOXO3
	Positive regulation of neuron apoptotic process	27.37	1,765E-04	ATM/FOXO3/NR3C1
	Regulation of stem cell differentiation	22.75	3,049E-04	EZH2/STAT3/YAP1
	Glutathione metabolic process	22.09	3,687E-03	G6PD/GSTM3
	Regulation of neuron apoptotic process	15.48	2,000E-06	ATM/CEBPB/FOXO3/G6PD/GDF5/NR3C1
	Neuroinflammatory response	15.17	7,662E-03	ATM/NR3C1
	Astrocyte differentiation	14.30	8,577E-03	NR3C1/STAT3
	Neuron apoptotic process	12.93	5,630E-06	ATM/CEBPB/FOXO3/G6PD/GDF5/NR3C1
	Neural precursor cell proliferation	11.31	2,313E-03	FOXO1/FOXO3/POU3F3
HOTAIR	Neuron projection arborization	30.26	1,981E-03	IGF2BP1/LRRK2
	Dopaminergic neuron differentiation	23.62	3,238E-03	FOXA1/VEGFA
	Regulation of dendritic spine morphogenesis	22.52	3,556E-03	LRRK2/PTEN
	Modulation of excitatory postsynaptic potential	19.76	4,595E-03	LRRK2/PTEN
	Regulation of neuroblast proliferation	18.27	5,357E-03	LRRK2/VEGFA
	Synaptic vesicle localization	17.61	5,758E-03	LRRK2/PTEN
	Hippocampus development	15.45	9,581E-04	EZH2/PTEN/SRF
	Positive regulation of axonogenesis	12.41	1,130E-02	SRF/VEGFA
	Regulation of synaptic transmission, glutamatergic	12.10	1,186E-02	CCL2/LRRK2
	Dendritic spine organization	11.95	1,215E-02	LRRK2/PTEN
	Regulation of neuron differentiation	9.88	6,961E-04	BMP7/FOXA1/LRRK2/PTBP1
	Regulation of axonogenesis	9.31	4,062E-03	PTEN/SRF/VEGFA
	Neural tube development	9.19	4,209E-03	BMP7/FOXA1/MTHFR
	Telencephalon development	8.80	2,422E-04	EZH2/IGF2BP1/LRRK2/PTEN/SRF
	Neuron migration	7.93	6,329E-03	SRF/STAT3/VEGFA
	Gliogenesis	6.99	6,917E-04	CCL2/EZH2/PTEN/STAT3/TLR4
	Regulation of neurogenesis	6.16	1,223E-03	BMP7/EZH2/PTEN/SRF/VEGFA
	Forebrain development	5.93	1,443E-03	EZH2/IGF2BP1/LRRK2/PTEN/SRF
	Regulation of nervous system development	5.17	2,628E-03	BMP7/EZH2/PTEN/SRF/VEGFA
MALAT1	Regulation of dendritic cell differentiation	42.73	9,710E-04	CEBPB/LILRB2
	Radial glial cell differentiation	39.68	1,130E-03	METTL3/STAT3
	Central nervous system myelination	21.36	3,925E-03	ID4/PTEN
	Axon ensheathment in central nervous system	21.36	3,925E-03	ID4/PTEN
	Regulation of stem cell differentiation	20.07	5,278E-07	EZH2/FGF2/LTBP3/METTL3/SOX9/STAT3

	Oligodendrocyte differentiation	15.72	2,236E-06	CTNNB1/ID4/PTEN/SOX9/SRSF1/SUZ12
	Regulation of dendritic spine morphogenesis	12.91	1,049E-02	CFL1/PTEN
	Glial cell differentiation	12.81	1,321E-10	CTNNB1/FGF2/ID4/LIN28A/METTL3/PTEN/SOX2/SOX9/SRSF1/STAT3/SUZ12/TLR4
	Autonomic nervous system development	12.07	1,194E-02	CTNNB1/RET
	Regulation of oligodendrocyte differentiation	12.07	1,194E-02	CTNNB1/ID4
	Gliogenesis	10.43	2,646E-10	CTNNB1/EZH2/FGF2/ID4/LIN28A/METTL3/PTEN/SOX2/SOX9/SRSF1/STAT3/SUZ12/TLR4
	Regulation of neuron differentiation	8.50	7,417E-05	FGF2/ID4/LIN28A/RET/SOX2/SOX9
	Regulation of neural precursor cell proliferation	8.16	5,943E-03	CTNNB1/FGF2/ID4
	Hindbrain development	7.03	2,533E-03	CTNNB1/EZH2/FGF2/GLI1
	Regulation of neuron apoptotic process	6.83	2,450E-04	CEBPB/CTNNB1/MYBL2/NONO/PARP1/SIRT1
	Forebrain development	5.80	1,885E-06	CTNNB1/E2F1/EZH2/FGF2/GLI1/ID4/ITGAM/METTL3/PTEN/SOX2
	Regulation of neurogenesis	4.24	2,885E-03	CTNNB1/EZH2/FGF2/ID4/LIN28A/PTEN
	Regulation of nervous system development	3.56	6,714E-03	CTNNB1/EZH2/FGF2/ID4/LIN28A/PTEN
**NEAT1**	Radial glial cell differentiation	45.73	8,521E-04	METTL14/STAT3
	Neuroendocrine cell differentiation	40.01	1,119E-03	BMP2/JAG1
	Myeloid dendritic cell activation	22.86	3,441E-03	HMGB1/TRAF6
	Response to amyloid-beta	11.85	1,237E-02	ICAM1/TLR4
	Glutathione metabolic process	8.35	1,371E-02	SLC7A11/SOD2
	Metencephalon development	6.56	5,579E-03	CDK5R1/EZH2/KAT2A
	Regulation of neuron apoptotic process	6.47	9,795E-04	CASP8/CDK5R1/EGR1/NONO/SOD2
	Gliogenesis	6.47	9,807E-05	BMP2/CDK5R1/EZH2/METTL14/SRSF1/STAT3/TLR4
	Glial cell differentiation	6.15	1,299E-03	BMP2/METTL14/SRSF1/STAT3/TLR4
	Hindbrain development	6.07	1,326E-02	CDK5R1/EZH2/KAT2A
	Telencephalon development	5.82	1,663E-03	BMP2/CDK5R1/EZH2/KAT2A/SLC7A11
	Forebrain development	5.49	2,702E-04	BMP2/CDK5R1/E2F1/EZH2/KAT2A/METTL14/SLC7A11
	Neuron apoptotic process	5.48	2,161E-03	CASP8/CDK5R1/EGR1/NONO/SOD2
	Regulation of synapse organization	5.26	6,994E-03	CDK5R1/MAPK14/NEDD4/SLC7A11
	Regulation of synapse structure or activity	5.14	7,610E-03	CDK5R1/MAPK14/NEDD4/SLC7A11
**MIAT**	Regulation of neuroinflammatory response	59,025	5,150E-04	IL1B/MMP9
	Neuroinflammatory response	42,66	4,397E-05	IL1B/JAK2/MMP9
	Glial cell activation	41,42	1,045E-03	IL1B/JAK2
	Astrocyte differentiation	26,82	2,468E-03	IL1B/STAT3
	Regulation of gliogenesis	21,08	3,961E-03	EZH2/IL1B
	Gliogenesis	10,23	2,858E-03	EZH2/IL1B/STAT3
	Regulation of neurogenesis	9,01	4,092E-03	EZH2/IL1B/YTHDF2
**MEG3**	Sympathetic nervous system development	57.23	5,523E-04	GATA3/SOX4
	Positive regulation of glial cell differentiation	27.37	2,417E-03	CXCR4/HDAC1
	Autonomic nervous system development	27.37	2,417E-03	GATA3/SOX4
	Axon regeneration	23.31	3,315E-03	JAK2/KLF4

	Regulation of stem cell differentiation	22.75	3,049E-04	EZH2/HDAC1/STAT3
	Neuron projection regeneration	20.64	4,211E-03	JAK2/KLF4
	Hippocampus development	20.09	4,396E-04	EZH2/HDAC1/PTEN
	Stem cell population maintenance	17.29	9,210E-06	FOXO1/HDAC1/KLF4/SOX4/STAT3
	Regulation of gliogenesis	16.86	7,333E-04	CXCR4/EZH2/HDAC1
	Regulation of glial cell differentiation	15.93	6,965E-03	CXCR4/HDAC1
	Response to axon injury	14.64	8,206E-03	JAK2/KLF4
	Glial cell development	14.20	1,206E-03	PTEN/SOX4/TLR4
	Neuron migration	10.32	2,996E-03	CXCR4/GATA3/STAT3
	Regulation of neuron projection development	6.84	7,223E-04	EZH2/GATA3/KLF4/MDM2/PTEN
	Forebrain development	6.17	3,769E-03	CXCR4/EZH2/HDAC1/PTEN
**PVT1**	Positive regulation of amyloid precursor protein catabolic process	38.74	1,209E-03	ROCK2/SP1
	Regulation of amyloid-beta formation	20.17	4,413E-03	ROCK2/SP1
	Cellular response to nerve growth factor stimulus	20.17	4,413E-03	EIF4A3/KAT2A
	Response to nerve growth factor	19.37	4,780E-03	EIF4A3/KAT2A
	Regulation of amyloid precursor protein catabolic process	17.61	5,758E-03	ROCK2/SP1
	Amyloid-beta formation	17.29	5,963E-03	ROCK2/SP1
	Vesicle docking	15.13	7,726E-03	VAMP3/YKT6
	Amyloid-beta metabolic process	14.90	7,961E-03	ROCK2/SP1
	Amyloid precursor protein catabolic process	14.24	8,684E-03	ROCK2/SP1
	Hindbrain development	12.26	3,086E-04	EZH2/HNF1B/KAT2A/NFIB
	Neuroinflammatory response	11.67	1,272E-02	JAK2/MMP9
	Neural tube closure	10.88	1,453E-02	KAT2A/TGFB1
	Glial cell differentiation	7.45	1,976E-03	STAT3/NFIB/SOX2/TGFB1
	Gliogenesis	6.99	6,917E-04	EZH2/STAT3/NFIB/SOX2/TGFB1
	Regulation of neurogenesis	4.82	8,547E-03	EZH2/FN1/TGFB1/YAP1
	Forebrain development	4.74	9,718E-03	EZH2/KAT2A/NFIB/SOX2

Table S5. Selection of main LncRNA-mediated Gene-Process Interactions related to the CNS in mouse. The processes for each lncRNA are ranked in descending order by their Fold Enrichment value.

LncRNA name	Biological Process	Fold Enrichment	*p-value*	Gene
**GAS5**	Bergmann glial cell differentiation	150.55	6,624E-03	Vim
	Regulation of mechanoreceptor differentiation	150.55	6,624E-03	Notch1
	Neuroendocrine cell differentiation	112.91	8,822E-03	Notch1
	Myelin maintenance	100.36	9,920E-03	Akt1
	Negative regulation of oligodendrocyte differentiation	100.36	9,920E-03	Notch1
	Positive regulation of dendrite development	82.12	1,211E-02	Ezh2
	Neuronal stem cell population maintenance	72.26	1,375E-02	Foxo1
	Regulation of glial cell proliferation	69.48	4,347E-04	Notch1/Vim
	Regulation of long-term neuronal synaptic plasticity	46.32	2,138E-02	Egr1
**HOTAIR**	Negative regulation of neuron migration	131,38	7,586E-03	Stat3
	Pyramidal neuron development	111.17	8,960E-03	Atg7
	Microglia differentiation	90.32	1,102E-02	Tgfb1
	Pyramidal neuron differentiation	80.29	1,239E-02	Atg7
	Regulation of neuron maturation	76.06	1,307E-02	Bcl2
	Regulation of motor neuron apoptotic process	72.26	1,375E-02	Bcl2
	Forebrain neuron development	48.17	2,056E-02	Atg7
	Central nervous system neuron axonogenesis	33.61	2,935E-02	Atg7
	Neuroblast proliferation	29.49	2,077E-03	Kdm1a/Tgfb1
	Regulation of gliogenesis	20.21	4,356E-03	Ezh2/Tgfb1
	Regulation of neuron apoptotic process	18.06	6,209E-05	Bcl2/Atg7/Tgfb2/Tgfb3
	Regulation of neuron differentiation	17.69	6,164E-04	Dnmt3b/Bcl2/Ptbp1
	Forebrain development	10.15	3,027E-03	Kdm1a/Atg7/Atg16l1
	Regulation of neurogenesis	8.77	4,556E-03	Ezh2/Kdm1a/Tgfb1
**MALAT1**	Regulation of oxidative stress-induced neuron intrinsic apoptotic signaling pathway	117.02	1,318E-04	Hif1a/Ctnnb1
	Synaptic vesicle clustering	111.17	1,464E-04	Pten/Ctnnb1
	Glial cell fate commitment	123.53	1,180E-04	Sox6/Ctnnb1
	Oligodendrocyte differentiation	27.12	1,820E-04	Pten/Sox6/Ctnnb1
	Cerebellum development	29.26	1,454E-04	Ezh2/Ulk1/Trp53
	Metencephalon development	26.06	2,047E-04	Ezh2/Ulk1/Trp53
	Regulation of neurogenesis	18.00	8,201E-09	Ezh2/Rela/Hif1a/Pten/Ulk1/Trp53/Kdr/Ctnnb1
	Neuron apoptotic process	17.51	9,289E-07	Bcl2l11/Hif1a/Birc5/Trp53/Kdr/Ctnnb1
	Neuroblast proliferation	34.03	9,284E-05	Hif1a/Trp53/Ctnnb1
	Neuron migration	16.03	8,451E-04	Satb2/Ulk1/Ctnnb1
	Neuron projection extension	16.03	8,451E-04	Pten/Ulk1/Ctnnb1
	Regulation of synapse organization	10.79	2,622E-03	Pten/Mapk14/Ctnnb1
	Synaptic transmission, GABAergic	31.76	1,810E-03	Ezh2/Pten
**NEAT1**	Regulation of oxidative stress-induced neuron intrinsic apoptotic signaling pathway	101.42	1,761E-04	Pink1/Ctnnb1

	Glial cell apoptotic process	96.35	1,956E-04	Trp53/Casp3
	Regulation of modification of postsynaptic actin cytoskeleton	96.35	1,033E-02	Ptk2
	Neuron intrinsic apoptotic signaling pathway in response to oxidative stress	91.76	2,160E-04	Pink1/Ctnnb1
	Regulation of synaptic transmission, dopaminergic	87.59	1,136E-02	Pink1
	Neuron fate determination	87.59	1,136E-02	Ctnnb1
	Negative regulation of neuron migration	87.59	1,136E-02	Bace1/Cxcr4
	Detection of mechanical stimulus involved in sensory perception of pain	83.78	2,599E-04	Bace1/Cxcr4
	Negative regulation of motor neuron apoptotic process	74.12	1,341E-02	Rock1
	Neural plate development	68.82	1,444E-02	Ctnnb1
	Midbrain dopaminergic neuron differentiation	68.82	1,444E-02	Ctnnb1
	Regulation of modification of postsynaptic structure	53.53	1,852E-02	Ptk2
**MIAT**	Noradrenergic neuron differentiation	578.10	1,729E-03	Sox4
	Glial cell activation	293.94	8,054E-08	Il1b/Tnf/Casp1
	Microglial cell activation	231.24	2,923E-05	Tnf/Casp1
	Regulation of glial cell proliferation	206.46	3,673E-05	Il1b/Tnf
	Glial cell development	82.58	2,307E-04	Il1b/Sox4
	Cerebellar cortex development	80.29	1,239E-02	Ezh2
	Gliogenesis	58.24	1,734E-07	Ezh2/Il1b/Tnf/Sox4
	Spinal cord development	56.67	1,752E-02	Sox4
	Glial cell differentiation	39.46	1,004E-03	Il1b/Sox4
	Regulation of neuron apoptotic process	36.13	1,195E-03	Il1b/Tnf
	Ensheathment of neurons	29.49	3,345E-02	Tnf
	Axon ensheathment	29.49	3,345E-02	Tnf
	Positive regulation of neuron projection development	24.18	4,067E-02	Ezh2
	Regulation of neuron differentiation	23.59	4,167E-02	Il1b
**MEG3**	Negative regulation of neuroblast proliferation	190.16	4,885E-05	Trp53/Tgfb1
	Microglia differentiation	190.16	4,885E-05	Myd88/Tgfb1
	Negative regulation of oxidative stress-induced neuron intrinsic apoptotic signaling pathway	126.78	7,861E-03	Atf4
	Bergmann glial cell differentiation	126.78	7,861E-03	Mapk1
	Postsynapse to nucleus signaling pathway	95.08	1,047E-02	Stat3
	Regulation of skeletal muscle cell proliferation	95.08	1,047E-02	Stat3
	Neuroepithelial cell differentiation	60.85	4,921E-04	Tgfb1/Smad2
	Regulation of glutamate secretion	58.51	1,696E-02	Il1b
	Negative regulation of oxidative stress-induced intrinsic apoptotic signaling pathway	58.51	1,696E-02	Atf4
	Positive regulation of glial cell differentiation	52.46	6,620E-04	Il1b/Tgfb1
	Astrocyte differentiation	48.04	3,208E-05	Il1b/Mapk1/Stat3
	Gamma-aminobutyric acid signaling pathway	47.54	2,083E-02	Atf4
	Regulation of skeletal muscle tissue development	47.54	2,083E-02	Tgfb1
**PVT1**	Microglia differentiation	225.82	4,420E-03	Myd88
	Regulation of synapse maturation	157.09	6,349E-03	Rock2
	Postsynaptic actin cytoskeleton organization	157.09	6,349E-03	Rock2
	Postsynaptic cytoskeleton organization	157.09	6,349E-03	Rock2
	Positive regulation of dendrite development	164.23	6,073E-03	Ezh2

Transport across blood-brain barrier	172.05	5,798E-03	Slc2a1
Gliogenesis	45.51	2,580E-08	Myc/Ezh2/Suz12/Eed/Myd88
Glial cell differentiation	36.99	5,560E-05	Suz12/Eed/Myd88
Regulation of gliogenesis	50.53	6,674E-04	Myc/Ezh2
Oligodendrocyte differentiation	58.75	4,946E-04	Suz12/Eed
Cortical actin cytoskeleton organization	86.03	1,157E-02	Rock2
Cerebellar cortex development	50.18	1,976E-02	Ezh2
Synaptic transmission, GABAergic	51.62	1,921E-02	Ezh2
Positive regulation of glial cell proliferation	120.44	8,274E-03	Myc
